# Skin-draining lymph node priming is sufficient to induce sterile immunity against pre-erythrocytic malaria

**DOI:** 10.1002/emmm.201201677

**Published:** 2012-12-19

**Authors:** Michel Obeid, Jean-François Franetich, Audrey Lorthiois, Audrey Gego, Anne Charlotte Grüner, Maurel Tefit, Claude Boucheix, Georges Snounou, Dominique Mazier

**Affiliations:** 1 Inserm, UMR-S 945Paris, France; 2 Faculté de Médecine Pitié-Salpêtrière, Université Pierre et Marie Curie-Paris 6, CHU Pitié-SalpêtrièreParis, France; 3 UMR 7206 Eco-anthropologie et Ethnobiologie, Département Hommes, Natures et Sociétés, Muséum National d'Histoire NaturelleParis, France; 4 Inserm, UMR-S 1004Villejuif, France; 5 Université Paris-Sud 11Villejuif, France; 6 AP-HP, Groupe Hospitalier Pitié-Salpêtrière, Service Parasitologie-MycologieParis, France

**Keywords:** anti-malaria vaccination, dendritic cells, liver-stage, pre-erythrocytic immunity, skin vaccination approach

## Abstract

The *Plasmodium*-infected hepatocyte has been considered necessary to prime the immune responses leading to sterile protection after vaccination with attenuated sporozoites. However, it has recently been demonstrated that priming also occurs in the skin. We wished to establish if sterile protection could be obtained in the absence of priming by infected hepatocytes. To this end, we developed a subcutaneous (s.c.) immunization protocol where few, possibly none, of the immunizing irradiated *Plasmodium yoelii* sporozoites infect hepatocytes, and also used CD81-deficient mice non-permissive to productive hepatocyte infections. We then compared and contrasted the patterns of priming with those obtained by intradermal immunization, where priming occurs in the liver. Using sterile immunity as a primary read-out, we exploited an inhibitor of T-cell migration, transgenic mice with conditional depletion of dendritic cells and adoptive transfers of draining lymph node-derived T cells, to provide evidence that responses leading to sterile immunity can be primed in the skin-draining lymph nodes with little, if any, contribution from the infected hepatocyte.

## INTRODUCTION

Vaccination leading to sterile protection against malaria infection was first obtained in 1967, when it was shown that immunization of mice with live, radiation-attenuated *Plasmodium berghei* sporozoites leads to full protection from a challenge with live sporozoites (Nussenzweig et al, 1967). The approach was further refined so that induction and long-term maintenance of sterile immunity against a *P. falciparum* sporozoite challenge could be obtained in all immunized hosts and crucially in humans (Clyde, 1975; Hoffman et al, 2002). The induced immunity is strictly stage-specific, in that it is solely effective against the pre-erythrocytic stages (PE, *i.e.* the sporozoite and the parasite's hepatic stages), with little if any inhibitory effect on the blood stages (Nussenzweig et al, 1969). The ability to induce full protection was later shown not to depend on the method used to attenuate the immunizing sporozoites, since chemical (Purcell et al, 2008a, b) or genetic (Mueller et al, 2005; van Dijk et al, 2005) attenuation are equally effective, but that the attenuated sporozoites used for immunization must be motile. Moreover, the efficacy of the induced sterile protection depended on the route of administration. Attenuated sporozoites inoculated intravenously (i.v.) or by mosquito bite (mb) consistently protected all recipients from a challenge infection (Chatterjee et al, 1999; Douradinha et al, 2007; Druilhe & Marchand, 1989; Richards, 1977; Spitalny & Nussenzweig, 1972), whereas intramuscular, intraperitoneal, subcutaneous (s.c.), or *per os* administration was less efficient (Douradinha et al, 2007; Druilhe & Marchand, 1989; Spitalny & Nussenzweig, 1972). The superiority of the i.v. route over the s.c. or intradermal (i.d.) routes has recently been confirmed (Epstein et al, 2011). Taken together, these observations implied that induction of full protection depends on immunizing attenuated sporozoites reaching the liver and infecting their host cell, the hepatocyte, where they can persist without maturing for extended periods of time (Chatterjee et al, 1999; Druilhe & Marchand, 1989; Mellouk et al, 1990; Mueller et al, 2007; Ramsey et al, 1982; Scheller & Azad, 1995; Silvie et al, 2002; van Dijk et al, 2005; Vanderberg et al, 1968).

The immune responses induced by attenuated sporozoites that lead to sterile protection were first thought to be antibody-mediated, because sera from immunized hosts are capable of blocking sporozoite motility (Stewart et al, 1986) and inhibiting sporozoite invasion *in vitro* (Hollingdale et al, 1982). The antibodies induced by immunization were shown to predominantly recognize the major constituent of the sporozoite surface, the circumsporozoite protein (CS), justifying the selection of the parasite antigen as a vaccine candidate. However, it was later shown that sterile protection was actually mainly due to CD8^+^ T-cell-mediated killing of *Plasmodium*-infected hepatocytes. This was demonstrated by antibody-mediated depletion of CD8 T cells and when adoptive transfer of a CD8^+^ T-cell clone specific to CS epitope fully protected mice from sporozoite challenge (Romero et al, 1989; Schofield et al, 1987; Weiss et al, 1988). However, the very low frequency of naïve precursors to malarial antigens primed by immunization hindered studies aimed at understanding the detailed mechanisms whereby protection is induced and maintained. The generation of transgenic mice expressing a T-cell receptor specific for an MHC-restricted murine malaria CS epitope (Sano et al, 2001) initiated an on-going series of elegant experiments that are contributing to elucidate the chain of events following attenuated-sporozoite immunization (Carvalho et al, 2002; Cockburn et al, 2010, 2011; Hafalla et al, 2002, 2003; Morrot et al, 2005).

The manner in which the protective immune responses are primed is of particular importance to the development of an effective anti-PE vaccine. Until recently, it was assumed that priming was principally mediated via the infected hepatocyte, which is consistent with the dependence of sterile protection on i.v. or mosquito bite inoculation of live sporozoites capable of invading and maturing in hepatocytes. This notion was underpinned by the assumption that sporozoites reach the liver via the blood stream within a very short time, and that those that fail are rapidly cleared from the skin, where they are deposited by the mosquito. However, recent studies revealed not only that a significant proportion of sporozoites actually remain in the skin for many hours, often draining to the lymph nodes (Amino et al, 2006; Yamauchi et al, 2007), but also that some can mature in the skin where they might remain for many days (Gueirard et al, 2010). This would provide sufficient time for the immune system to interact with the sporozoites in the skin, given that antigen persistence was shown to lead to optimal CD8^+^ responses (Cockburn et al, 2010). These observations and others (Cockburn et al, 2011) raised doubts about the liver as the exclusive priming site, where dendritic cells (DC) acquire antigens from infected hepatocytes. It was then shown that cutaneous lymph node DC are also capable of priming CD8^+^ T cells that would then contribute to inhibit the hepatic parasite (Chakravarty et al, 2007). This elegant demonstration relied on the use of an adoptively transferred TCR transgenic CD8^+^ T-cell clone specific to a defined CS epitope. However, the actual contribution of skin-primed immune responses to sterile immunity could not be deduced from this study. Protection was only measured in terms of a reduction of the number and development of the hepatic parasites following challenge, without demonstrating that the challenged mice failed to develop a blood stage infection, *i.e.* sterile immunity. Indeed, a few surviving hepatic parasites are sufficient to lead to an infection with its consequent pathology. Moreover, the experiments relied on transfer of a large CD8^+^ T-cell clone (1 million cells), a number that is far above that observed following immunization with attenuated sporozoites. Subsequently, protocols based on i.d. or s.c. immunization were shown to lead to high levels of protection (Butler et al, 2011; Inoue & Culleton, 2011; Voza et al, 2010). Nonetheless, the immunization protocols in these four studies could not exclude a role for priming at sites distant from the skin, and in particular by antigens from hepatocytes that the immunizing sporozoites infect and in which they develop.

We wished to establish whether priming by vaccination with irradiated sporozoites under conditions where few, if any, hepatocytes are infected, would be sufficient to induce sterile protection. In order to address this question, we adopted two immunization protocols where exposure to the hepatic stages is minimized and analysed the resulting cellular immune responses. We provide evidence that priming by irradiated sporozoites occurring primarily in the skin-draining lymph node can confer sterile protection against challenge with normal sporozoites.

## RESULTS

### Long-lasting sterile pre-erythrocytic stage immunity can be elicited by subcutaneous immunization

The route of sporozoite inoculation is known to modify the proportion of hosts that will develop blood stage infections, *i.e.* the number of productive hepatic infections. For i.v. inoculation, a minimum of 5000 *P. yoelii* 265BY (PyWT) sporozoites will ensure a blood stage infection in all inoculated mice. We noticed in preliminary observations that s.c. inoculation of 1000–40,000 PyWT sporozoites did not generally lead to blood infection unless bleeding at the site of injection occurred (this can be minimized with practice), whereas i.d. inoculation with similar numbers of PyWT sporozoites invariably led to blood stage infection. Thus, liver stage development is minimal or does not occur following s.c. inoculation, presumably because the sporozoites do not exit the skin or they have lost their infectivity by the time they do.

We then conducted a series of immunization experiments using different methods of administration to establish the immunization doses required to induce sterile protection against challenge with infectious sporozoites (PyWT). We first tested the classic *P. yoelii* 265BY irradiated-sporozoites (γ-spz) immunization protocol, of a primary dose of 75 × 10^3^ γ-spz followed by four booster doses of 20 × 10^3^ γ-spz at 2-weekly intervals (Nussenzweig et al, 1967), administered i.v., i.d. or s.c. Seven days following the last booster immunization the mice were challenged i.d., i.v. or by mb with PyWT sporozoites. Whereas i.v. immunization elicited sterile protection in nearly all the mice, only few of the i.d.- or s.c.-immunized mice (13–20%) were protected (Table 1). Nonetheless, full protection of all the mice could be obtained for the i.d. and s.c. routes by increasing the immunization dose to 100 × 10^3^ γ-spz administered on three occasions at a weekly interval (Table 1), and we adopted this as the standard immunization protocol. Lower immunizing doses (100 × 10^3^ given once or twice separated by 1 week) were effective for the i.v. route but not for the s.c. or i.d. routes (Table 1). Higher doses administered twice at a weekly interval (200 × 10^3^), or just once (600 × 10^3^) induced full protection for the i.v. and s.c. route, but only protected 60–80% of the mice for the i.d. route (Table 1). It should be noted that in our s.c. immunization protocols, the number of irradiated *Plasmodium yoelii* sporozoites needed to achieve sterile protection was higher than that required for the same purpose in other studies: 42,000–60,000 irradiated *P. yoelii* sporozoites (Epstein et al, 2011), 150,000 genetically attenuated *P. yoelii* sporozoites (Butler et al, 2011) or 60,000 followed by a boost with 30,000 irradiated *P. yoelii* sporozoites (Voza et al, 2010). This most probably reflects the fact that the sporozoites from the *P. yoelii* 265BY line are 100 times less infectious to BALB/c mice than those of the *P. yoelii* 17XNL line (Belnoue et al, 2004) that was used in the three studies above. We then investigated the longevity of sterile protection in our vaccination model. For all three groups (i.v., i.d. or s.c. immunization), mice that were fully protected against a first challenge were re-challenged i.d., i.v. or by mb, 6 months later, with 5 × 10^3^ PyWT, and none developed blood stage infection (Table 1). Furthermore, another three groups of immunized mice (i.v., i.d. or s.c.) were only challenged 6 months after the last booster immunization boost, and again none developed a patent blood stage infection (Table 1).

**Table 1:** Sterile protection in BALB/c mice immunized with attenuated sporozoites

Immunogen	Immunizing dose (×10^3^)	Immunization route (days between 2 boosts)	Challenge route	Challenge dose (days after last immunization)	No. protected/no. challenged	Protection (%) (mean ± SD)
PBS	75/20/20/20/20	i.v. (14), i.d. (14), s.c. (14)	i.v., i.d. or mb	5000 (7)	0/15, 0/15, 0/15	0, 0, 0
	75/20/20/20/20	i.v. (14)	i.v., i.d. or mb	5000 (7)	29/30, 29/30, 29/30	97 ± 6, 97 ± 6, 97 ± 6
	75/20/20/20/20	i.d. (14)	i.v., i.d. or mb	5000 (7)	5/30, 4/30, 4/30	17 ± 6, 13 ± 6, 13 ± 6
	75/20/20/20/20	s.c. (14)	i.v., i.d. or mb	5000 (7)	5/30, 5/30, 6/30	17 ± 6, 17 ± 6, 20 ± 0
γ-spz	100/100/100	i.v. (7), i.d. (7), s.c. (7)	i.v., i.d. or mb	5000 (7)	15/15, 15/15, 15/15	100, 100, 100
	100/100/100	i.v. (7), i.d. (7), s.c. (7)	i.v., i.d. or mb	5000 (180)	15/15, 15/15, 15/15	100, 100, 100
	100/100/100	i.v. (7), i.d. (7), s.c. (7)	i.v., i.d. or mb	5000 (7 + 180)	15/15, 15/15, 15/15	100, 100, 100
	100	i.v., i.d., s.c	i.d.	5000 (7)	15/15, 0/15, 0/15	100, 0, 0
	100/100	i.v. (7), i.d. (7), s.c. (7)	i.d.	5000 (7)	15/15, 0/15, 0/15	100, 0, 0
	200/200	i.v. (7), i.d. (7), s.c. (7)	i.d.	5000 (7)	15/15, 9/15, 15/15	100, 60 ± 0, 100
	600	i.v., i.d., s.c	i.d.	5000 (7)	15/15, 12/15, 15/15	100, 80 ± 0, 100

Attenuated sporozoites (γ-spz) were obtained by exposure to 80 Gy of γ-radiation from a ^137^Cesium source. Mice were challenged with 5 × 10^3^ PyWT (*P. yoelii* 265BY). Each immunization group had a naïve control group of five mice (*n* = 5) immunized with PBS. All control mice became patent on days 4 or 5 after PyWT challenge. Blood-stage patency was monitored three times a week by evaluation of Giemsa-stained blood smears from day 4 to day 14 post sporozoites infection. Results expressed as mean ± standard deviation (SD). All experiments were repeated three times (*n* = 3) with 5 or 10 mice in each group. mb, mosquito bite; i.d., intradermal; i.v., intravenous; s.c., subcutaneous.

### Infected hepatocytes are not involved in sterile immunity induced by subcutaneous immunization

**Figure 1:**
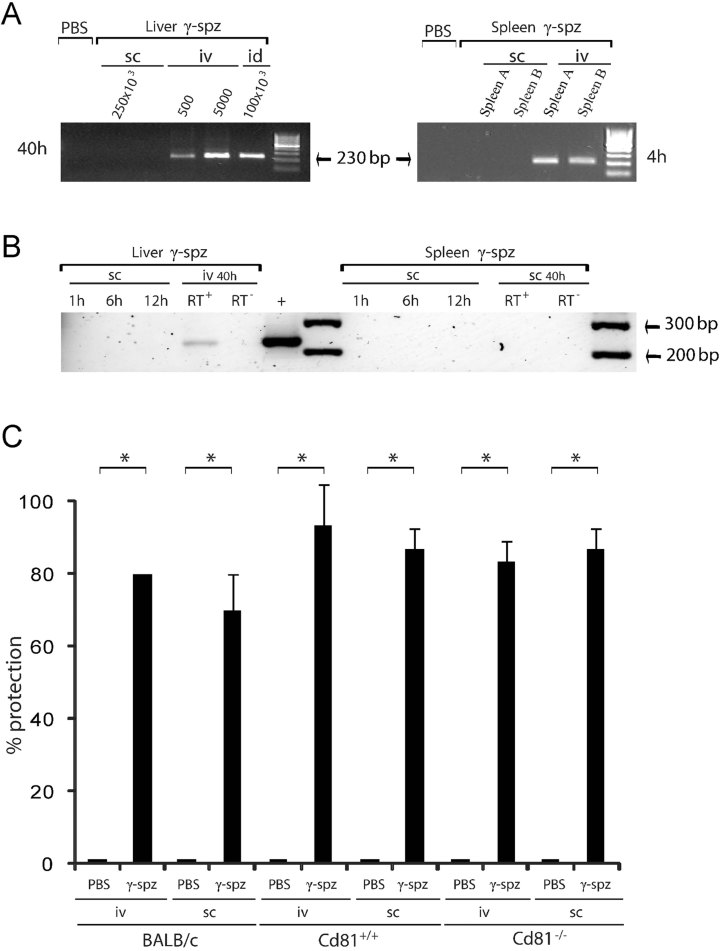
Sterile protection acquired by γ-spz immunization regimens associated with undetectable hepatic parasites RT-nPCR analysis of cDNA derived from total RNA purified from liver or spleen samples collected from mice inoculated with γ-spz s.c. (250 × 10^3^), i.d. (100 × 10^3^), or i.v. (5 × 10^2^ or 5 × 10^3^), 4 h post inoculation for the spleen and 40–44 h post-inoculation for the liver.Subcutaneous with 100 × 10^3^ γ-spz 1, 6, 12 or 40 h post inoculation or i.v. with 5 × 10^3^ γ-spz 40–44 h post inoculation. RT^−^ is a control for DNA contamination prepared in the absence of the reverse transcriptase. The + is a positive control for the amplification reaction from genomic *P. yoelii* 265BY DNA. Control mice received PBS.BALB/c, *Cd81*^+/+^, or *Cd81*^−/−^ mice were immunized s.c. or i.v. with PBS (controls) or three doses of 100 × 10^3^ γ-spz at 1-weekly interval. Five days after the last immunization, 30 × 10^6^ splenocytes were transferred into each naïve mice, and these were challenged 2 days later by i.d. inoculation with 5 × 10^3^ PyWT. The naïve recipients adoptively transferred with the splenocytes of control mice became patent at day 4–5 after PyWT challenge. Blood stage parasites could not be detected in any of the naïve recipients of splenocytes from immunized animals in Giemsa-stained blood smears collected three times a week from days 4 to 14 post sporozoite challenge. Results expressed as mean ± standard deviation (SD). All experiments were repeated three times (*n* = 3) with 10 mice per group at each time. *p = 0.0005 by Kruskal–Wallis test with Dunn's post-test.

It is possible that some of s.c.-inoculated attenuated sporozoites escape the skin and reach the liver, where they, or antigens derived from them, would also induce immune responses leading to sterile protection. Therefore, we used a sensitive amplification protocol to establish whether parasites could be detected in the spleens or livers of mice inoculated with 100 × 10^3^ γ-spz i.d., or with a substantially larger dose (250 × 10^3^) s.c. The spleens and livers of some experimental animals were harvested 4 or 40 h post-inoculation, respectively. In other s.c. inoculated animals, the spleens and livers were harvested at 1, 6, 12 and 40 h post-inoculation. Total RNA was isolated from these livers and spleens and used in an RT-nested PCR assay to detect small amounts of the parasite's small subunit ribosomal RNA gene. No amplification product was obtained in any of the liver or spleen samples collected from mice inoculated s.c. (Fig 1B), in contrast to robust amplification in the samples collected from i.d.- and i.v.-inoculated mice (Fig 1A). The sensitivity of the method is attested by the positive results observed for the liver samples obtained from mice inoculated i.v. with only 500 γ-spz (assuming most of the sporozoites invaded hepatocytes and that each parasite harbours a conservative 500 ribosomes, the aliquot assayed would correspond to about 150 target copies). Thus, it is likely that none, or substantially <0.2% of the 250 × 10^3^ γ-spz inoculated s.c. would have reached, or persisted in, the liver. Given that an i.v. immunization schedule based on 500 γ-spz does not lead to sterile protection against normal challenge, a minute proportion of the s.c.-inoculated γ-spz that might have made their way out of the skin to liver would not account for the protective immunity we obtained in our model.

In order to exclude a role for any attenuated parasites that might have developed intrahepatically, we repeated the immunizations in CD81-deficient mice (*Cd81*^−/−^). Indeed, it has previously been demonstrated that the expression of CD81 on the plasma membrane of hepatocytes is required for development of a productive *P. yoelii* hepatic infection (Silvie et al, 2003). Thus, *Cd81*^−/−^ mice inoculated i.v. with 10^6^ infectious PyWT failed to develop a blood stage infection (5000 PyWT is the minimum dose to obtain 100% blood stage infection in BALB/c mice). We immunized groups of BALB/c, *Cd81*^−/−^ or *Cd81*^+/+^ mice by γ-spz inoculated either s.c. or i.v., and subsequently harvested and adoptively transferred splenocytes from these mice into groups of naïve mice, which were challenged with the standard amount of 5 × 10^3^ PyWT sporozoites. The splenocytes adoptively transferred from all groups of immunized mice conferred strong protection against challenge in the recipient naïve mice (Fig 1C).

### The immunity induced by subcutaneous vaccination is primed in the skin-draining lymph nodes

Absence of CD81 on hepatocytes merely prevents productive infections but it does not alter the capacity of sporozoites to traverse hepatocytes (Silvie et al, 2003). Thus, it could be argued that the priming that led to sterile immunity was in part due to antigens derived from sporozoites in traversed (but not productively infected) hepatocytes. These antigens might then be secreted in the hepatocytes' cytoplasm for class I presentation, or out of the hepatocytes to hepatic lymph nodes via lymph or to the spleen via blood for class II presentation or induction of T cells by cross-priming. In order to explore the likelihood that such scenarios for alternative priming sites could account for the immunity observed (Fig 1C), the contribution of the liver and spleen to protection was explored.

**Figure 2:**
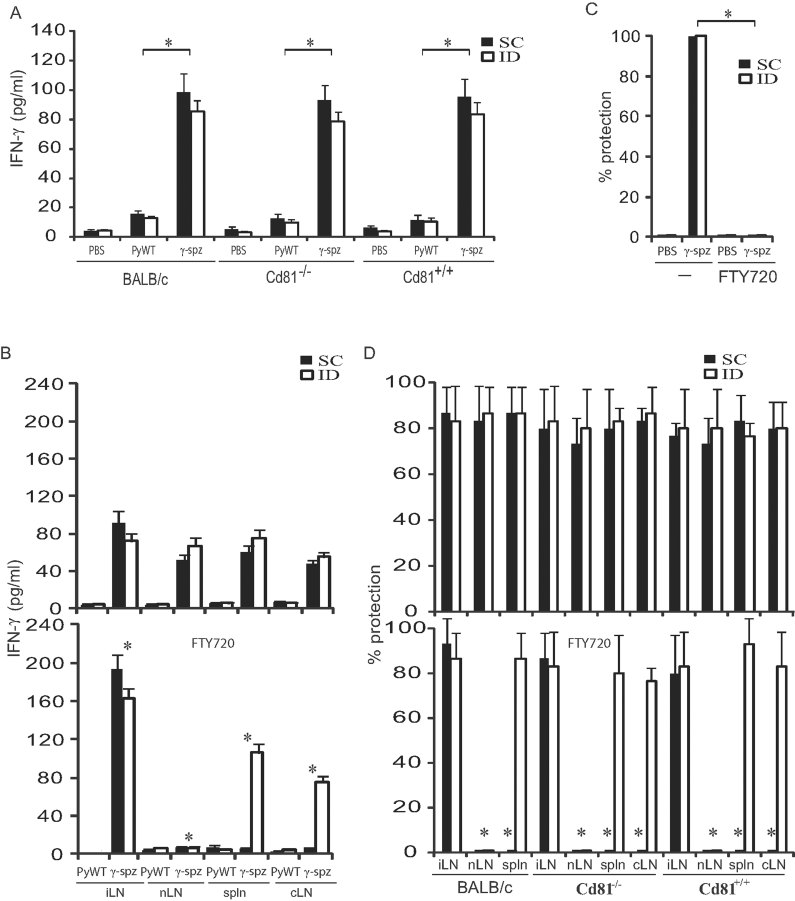
Investigations on the priming site in immunized animals Results expressed as mean ± standard deviation (SD). All experiments were repeated three times (*n* = 3) with five mice per group at each time. *p < 0.0001 for (**A**) and (**B**), 0.0017 for (**C**) or 0.0002 for (**D**) by Kruskal–Wallis test with Dunn's post-test. In (**B**) and (**D**) the * refer to statistical significance between data in the upper and lower panels. The level of T-cell priming following s.c. or i.d. γ-spz immunization (three 100 × 10^3^ doses at weekly intervals) of BALB/c, *Cd81*^+/+^, or *Cd81*^−/−^ mice (s.c. or i.d.), was assessed by measuring IFN-γ production subsequent to stimulation with a CS T-cell epitope, by cells isolated 4–5 days following the last immunizing dose from the right iLN draining the immunization site. Mice infected with PyWT sporozoites served as controls.In a separate experiment where the mice were immunized as above, the level of T-cell priming was similarly measured in cells isolated from iLN, their nLNs, the cLN, or from the spleen (spln). This was conducted for cells collected from animals that were untreated (upper panel) or FTY720-treated (lower panel) throughout the experiment.Mice were immunized s.c. or i.d. (two doses of 200 × 10^3^ γ-spz at 1 weekly interval, or PBS for controls) while under FTY720 treatment or not. Protection (undetectable blood stage parasites between days 4 and 14 post-challenge) was assessed after an i.v. challenge with 5 × 10^3^ PyWT.30 × 10^6^ cells isolated from the iLN, nLN, cLN or spln of BALB/c, *Cd81*^+/+^, or *Cd81*^−/−^ mice 5 days after immunization s.c. or i.d. with two doses of 200 × 10^3^ γ-spz at 1-weekly interval, were adoptively transferred into each of the naïve recipient mice, which were challenged i.d. (5 × 10^3^ PyWT sporozoites) 2 days later. The experiment was conducted for mice that were untreated (upper panel) or treated with FTY720 (lower panel) throughout the experiment. All recipient mice adoptively transferred with control mice splenocytes became patent by days 4 or 5 after PyWT challenge. Protection from challenge in the experimental animals was measured as above.

First, we assessed T-cell priming through the production of IFN-γ after stimulation by a specific parasite antigen, at different sites in i.d.-immunized mice, where priming is expected to occur concomitantly in the skin, the liver and the spleen, and in s.c.-immunized mice, where priming is expected to occur principally, or entirely, in the skin. Groups of BALB/c, *Cd81*^−/−^ or *Cd81*^+/+^ mice were immunized s.c. or i.d. with γ-spz. Cells were harvested from different sites for the IFN-γ assays. iLN cells from either s.c.- or i.d.-immunized mice showed strong IFN-γ production, following stimulation with a CS epitope, which contrasted with the weak IFN-γ production by iLN cells isolated from PyWT infected mice (Fig 2A). However, activated T cells could leave the priming site and circulate to other lymph nodes and organs within few days (4–5 days) after i.d. vaccination (Chakravarty et al, 2007). Consequently, we additionally sought the presence of activated T cells in the contra-lateral non-draining inguinal-lymph node (nLN), the celiac-lymph node (cLN) and the spleen. Five days after s.c. or i.d. vaccination with γ-spz, IFN-γ-producing T cells were equally present in the iLN, nLN, cLN and the spleen (Fig 2B). This could either be due to migration of activated T cells from the priming site in the skin (iLN) to the nLN, cLN and the spleen, or due to the presence of multiple priming sites. In order to distinguish between the two possibilities, migration of T lymphocytes out of the priming site was inhibited. This was achieved by treating mice with the immunosuppressive drug FTY720, which downregulates the expression of sphingosine-1-phosphate receptors (S1PRs) on the T lymphocyte surface, thereby preventing lymphocytes from migrating along a S1P gradient and reducing the T-cell egress from draining-lymph nodes (Brinkmann et al, 2010). In the i.d.- or s.c.-vaccinated and FTY720-treated mice, the level of activated T cells in the iLN increased about twofolds in contrast to the nLN, where activated T cells were no longer found (Fig 2B). Furthermore, treatment with FTY720 also reduced the level of activated T cells in the cLN and the spleen to negligible levels in the s.c.-immunized mice but not in the i.d.-immunized mice, where their levels increased about 1.5-fold (Fig 2B). These data suggest that whereas the iLN, CLN and the spleen are all priming sites in i.d.-immunized mice, in s.c.-immunized mice priming occurs mainly, if not exclusively, in the skin-draining lymph nodes (iLN).

Treatment with FTY720 results in the sequestration of lymphocytes in the iLN. Thus, if the protective T cells were solely primed at the level of the skin, then FTY720 treatment would also result in the absence of activated T cells in spleen, cLN and nLN. Consequently, mice immunized s.c. with γ-spz would fail to become protected against an infectious i.v. challenge, and the adoptive transfer of T cells purified from the nLN, cLN or the spleen of the immunized mice would not longer be able to protect naïve recipient mice from challenge. Indeed, none of the FTY720-treated mice immunized with γ-spz s.c. or i.d. resisted the standard i.v. challenge with PyWT sporozoites (Fig 2C). Moreover, T cells isolated from the non-draining lymph nodes nLN, cLN and the spleens of FTY720-treated BALB/c, *Cd81*^−/−^ or *Cd81*^+/+^ mice immunized s.c. with γ-spz were incapable to confer protection by adoptive transfer. In contrast, adoptively transferred cells isolated from the lymph nodes of untreated mice protected recipient mice against challenge (Fig 2D). The adoptive transfer of cells purified from the cLN or the spleen of FTY720-treated and i.d. vaccinated mice to naïve mice fully protected them against challenge, but not when the adoptively transferred cells were isolated from the nLN (Fig 2D).

### DC are required for the priming of protective immunity in s.c.-immunized mice

Dendritic cells play a crucial role in initiating primary cellular and humoral immune responses (Banchereau & Steinman, 1998; Kupper & Fuhlbrigge, 2004), and we wished to explore their contribution in our vaccination model. To this end, we employed transgenic mice expressing the diphtheria toxin (DT) receptor under the control of the CD11c promoter, such that injection of diphtheria toxin (DT) depletes all the CD11c^hi^ DC subsets for a short period of time, while epidermis-resident CD11c^low^ DC are relatively unaffected (Jung et al, 2002).

**Figure 3:**
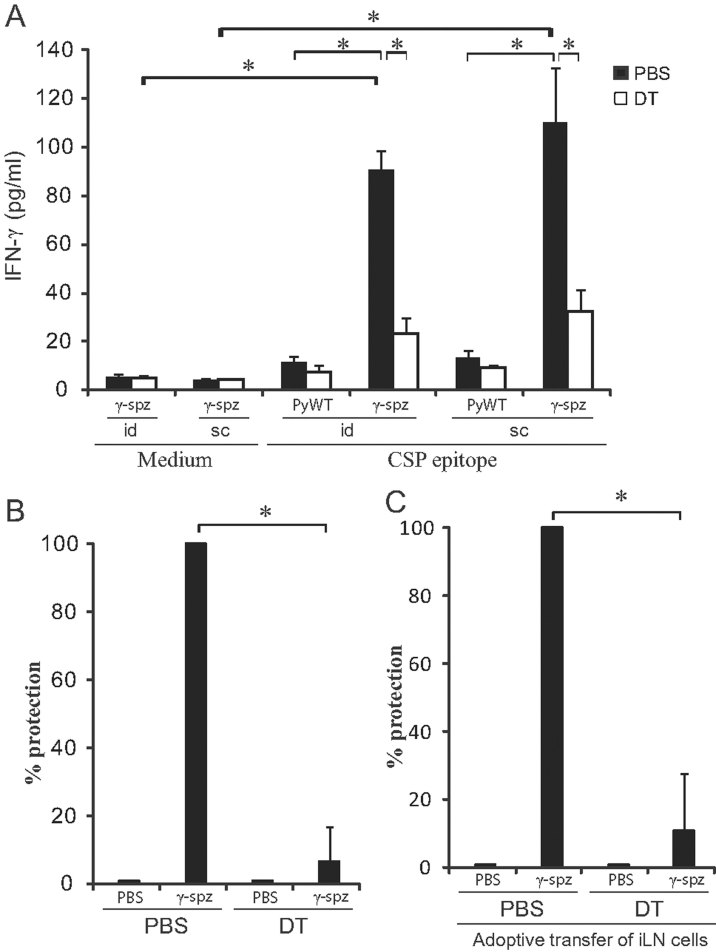
Dendritic cells mediate s.c.-primed immunity Results expressed as mean ± standard deviation (SD). All experiments were repeated three times (n = 3) with 3 to 5 mice per group at each time. * *p* = 0.0005 for (**A**), *p* = 0.023 for (**B**) and (**C**) by Kruskal–Wallis test with Dunn's post-test. BALB/c transgenic (CD11c-DTR) mice treated or not with DT to deplete DC, were immunized s.c. or i.d. with 200 × 10^3^ 265BY PyWT or γ-spz. The level of T-cell priming in the iLNs was then assessed through measurement of interferon-γ production after stimulation with a CS T-cell epitope.Assessment of the influence of DC depletion (by DT treatment, 2 days prior to each immunization) on the acquisition of sterile protection in BALB/c transgenic (CD11c-DTR) mice by s.c. immunization with two doses of 200 × 10^3^ γ-spz at 1-weekly interval. The mice were challenged i.d. 1 week after the last s.c. immunization with 5 × 10^3^ 265BY PyWT, and protection defined as the absence of blood stages in Giemsa-stained blood smears collected from day 4 to day 14 post sporozoites infection.Adoptive transfer of cells purified from the iLN of BALB/c transgenic (CD11c-DTR) mice whose DC were depleted or not (DT or PBS treatment), prior to a single immunization with 600 × 10^3^ γ-spz. The adoptively transferred cells were collected 5 days post-immunization and the recipient naïve mice were challenged 2 days later i.v. with 5 × 10^3^ PyWT and the appearance of blood-stage parasites was monitored as above.

In order to minimize the circulation of activated T cells that is likely to occur with repeated boosting doses, we adopted an effective vaccination regimen of a single s.c. inoculation 600 × 10^3^ γ-spz (Table 1). Groups of mice were immunized s.c. or i.d. with γ-spz, and one set was treated with DT whereas the other served as control. Cells from the iLN of control s.c.- or i.d.-immunized mice (*i.e.* not CD11c-depleted) produced high levels of interferon-γ in response to stimulus by a CSP epitope. In contrast, iLN cells from DT-injected immunized mice (*i.e.* CD11c DC-depleted) did not produce interferon-γ in response to the same stimulus (Fig 3A). Furthermore, the sterile protection conferred by immunization was completely abrogated by CD11c DC depletion (Fig 3B). Finally, adoptive transfer of iLN cells from control immunized mice protected recipient naïve mice against PyWT sporozoite challenge, but the iLN cells from CD11c DC-depleted immunized mice failed to do so (Fig 3C).

### Antigens in the immunizing sporozoites are sufficient to account for protective immunity

**Figure 4:**
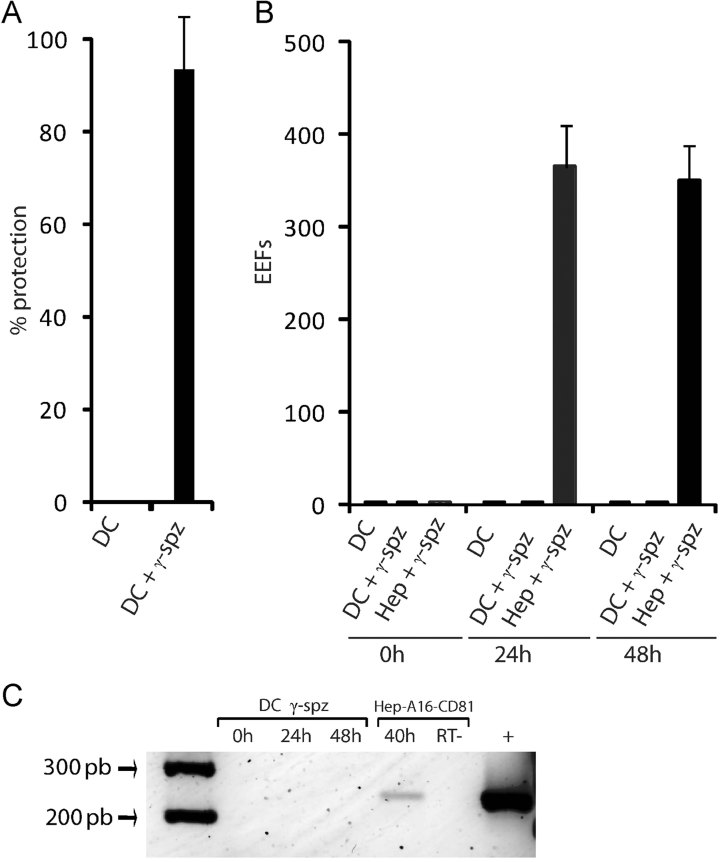
Protective antigens are probably present in the immunizing sporozoites All experiments were repeated three times (*n* = 3) with five mice per group (**A**) or by three triplicates (**B**) at each time. 100 × 10^3^ CD11c^+^-DC were sorted by FACS after co-culture with γ-spz or medium for 1.5 h, and then injected i.v. into recipient naïve mice. One week later, the recipient mice were challenged i.v. with 5 × 10^3^ PyWT and the appearance of blood stage parasites was monitored three times a week by microscopic examination of Giemsa-stained blood smears from day 4 to day 14 post sporozoites infection. Each immunization group had a naïve control group of five mice (*n* = 5) immunized with PBS.Exo-erythrocytic forms (EEFs) were detected by fluorescence microscopy after double staining with anti-CSP and anti-HSP70, in the FACS-purified DC after co-culture with γ-spz sporozoites (or medium) for 0, 24 or 48 h after the co-culture of DC. The infectiousness of the γ-spz was assessed in control wells containing primary mouse hepaotcyte (Hep). Results are expressed as mean ± standard deviation (SD).Parasites were also detected by RT-nPCR after co-culture of γ-spz and DC, in the DC fraction purified by FACS immediately, 24 or 48 h after the co-culture. The control for sporozoite infectiousness was HepG2-A16-CD81, which are highly receptive to *P. yoelii* sporozoites (Yalaoui et al, 2008). RT^−^ is a control for DNA contamination prepared in the absence of the reverse transcriptase. + is a positive control for the amplification reaction from genomic *P. yoelii* 265BY DNA.

In order to provide unequivocal evidence that the priming leading to sterile protection can be solely due to antigens present in the immunizing sporozoite, *in vitro*-generated DC were co-cultured with γ-spz for 90 min, after which the CD11c^+^-DC were FACS sorted and injected into recipient naïve mice. One week later, the recipient mice were challenged with 5 × 10^3^ PyWT i.v. None of the recipient mice developed a blood stage infection, *i.e.* the transfer of CD11c^+^-DC primed with γ-spz was sufficient to confer sterile protection against a sporozoite challenge (Fig 4A). Given the observations on extra-hepatic development of exo-erythrocytic forms (EEFs), we analysed the presence of EEFs in DC by a double immunofluorescence staining with anti-CSP and anti-HSP70 (two parasites markers). None were detected in the purified DC co-cultured with γ-spz that were maintained for 0, 24 or 48 h (Fig 4B). Additional confirmation was obtained by RT-nPCR assays on samples collected at the same time intervals, which also proved negative (Fig 4C). In order to demonstrate that the γ-spz batch employed in these experiments was capable of developing into EEFs, the co-culture and incubations were conducted in parallel using primary mouse hepatocytes (Fig 4B) or HepG2-A16-CD81 cells (Fig 4C). These were invaded by γ-spz, which then developed into recognizable and PCR-detectable EEFs (Fig 4B and C). Finally, when DC incubated with PyWT sporozoites were FACS-purified and inoculated into mice, these did not develop a blood stage parasitemia, demonstrating that viable sporozoites are unlikely to be retained with the FACS-purified DC.

### The role of immune effectors in the induction and expression of sterile immunity conferred by s.c. immunization

**Figure 5:**
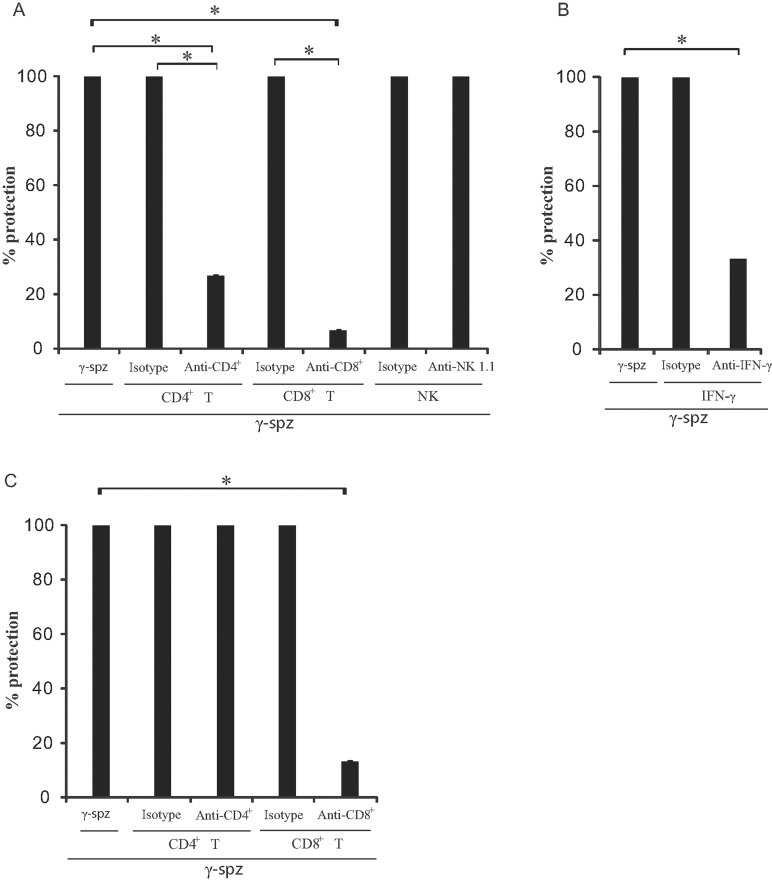
Characterization of the immune effectors implicated in the development of skin-primed pre-erythrocytic immunity Results expressed as mean ± standard deviation (SD). All experiments were repeated three times (*n* = 3) with five mice per group at each time. *p = 0.0052 for (**A**), 0.0211 for (**B**) or 0.0078 for (**C**) by Kruskal–Wallis test with Dunn's post-test. CD4^+^ T, CD8^+^ T or NK cells were depleted in BALB/c mice by *in vivo* injection of neutralizing antibodies 3 and 1 days before s.c. immunization with γ-spz (three doses of 100 × 10^3^ at 1-weekly intervals). The depletion was repeated once a week time before each immunization. The depletion of CD4^+^ T was 82% and that of CD8^+^ T was 86%. One week after the last immunization dose, the mice were challenged i.d. with 5 × 10^3^ PyWT, and the appearance of blood stage parasites monitored was monitored three times a week by evaluation of Giemsa-stained blood smears from day 4 to day 14 post sporozoites infection.Interferon-γ in BALB/c mice was neutralized by *in vivo* injection of antibodies 3 and 1 days before s.c. immunization as above. The depletion was repeated before each immunization dose. One week after the last dose, the mice were challenged i.d. with 5 × 10^3^ PyWT and blood stage patency was monitored as above.CD4^+^ or CD8^+^ T cells were depleted in γ-spz s.c. immunized BALB/c mice by *in vivo* injection of antibodies 1 week after the last s.c. γ-spz immunization and 3 days before the i.d. challenge with 5 × 10^3^ of PyWT, and blood stage patency was monitored as above.

We assessed the contribution of various immune system effectors to the induction of sterile immunity resulting from s.c. immunization with γ-spz. First, we explored the impact of depletion of CD4^+^ or CD8^+^ T cells, or NK cells during the induction phase. Sterile immunity was not induced by γ-spz immunization in the absence of CD8^+^ or CD4^+^ T cells, whereas the absence of NK cells did not interfere with the acquisition of sterile immunity (Fig 5A). Interferon-γ-neutralization significantly decreased the proportion of mice that became fully protected from sporozoite challenge (Fig 5B).

Although the presence of both CD4^+^ and CD8^+^ T cells was found to be crucial for the induction phase, depletion of CD8^+^, but not CD4^+^ T cells after s.c. immunization of mice with γ-spz abrogated the protection, indicating that only CD8^+^ T cells are required for the effector phase (Fig 5).

## DISCUSSION

The long-held perception that the priming necessary to generate the immunity conferred by vaccination with attenuated sporozoites occurs when the parasites reach and then develop in the liver had to be revisited when it was demonstrated that priming could also occur in the skin (Chakravarty et al, 2007). The concurrent discovery that some sporozoites deposited by the infected mosquito actually remain in the skin for extended periods of time (Amino et al, 2006; Yamauchi et al, 2007), where they might indeed initiate development (Gueirard et al, 2010), implicated the skin as a site of potential importance for inducing immunity against the pre-erythrocytic stages of *Plasmodium*. Indeed, it has been suggested that the skin stages might play an important immunomodulatory role in the acquisition of immunity against malaria, both under natural and artificial conditions; a hypothesis of some importance to vaccine development (Guilbride et al, 2012). This concept extends to the acquisition of immunity in other parasitic infections where the pathogens gain entry via the skin (Dominguez et al, 2012).

Protocols for immunization via the dermal or s.c. routes leading to high levels of protection were subsequently developed (Butler et al, 2011; Epstein et al, 2011; Inoue & Culleton, 2011; Voza et al, 2010). Nonetheless, the immunizing sporozoites used in these studies reached the liver where they infected and then developed in hepatocytes. The aim of the present work was to ascertain whether it would be possible to induce sterile immunity by vaccination with irradiated sporozoites (the gold standard anti-malarial vaccine protocol) if the contribution of hepatic stage parasite were excluded as a source of antigens to prime the protective immune responses. In order to address this question, it is necessary to dispose of experimental models where hepatic parasites do not occur despite inoculation with infectious sporozoites. We had previously noted that mice carefully inoculated with sporozoites subcutaneously, but not i.d., do not usually develop blood stage infection, presumably because few if any leave the skin to reach the liver. Furthermore, this can also be achieved in a mouse model deficient in CD81 where hepatocytes are non-permissive to productive infection (Silvie et al, 2003). We based our experiments on these models.

Having established a γ-spz-subcutaneous immunization protocol that induces long-lasting sterile immunity in BALB/c mice (irrespective of the route of challenge), we showed that few, if any, of the s.c. inoculated γ-spz reached the spleen or the liver. Sterile immunity was also obtained by s.c. immunization in *Cd81*^−/−^ mice (C57BL/6), where productive hepatic infections do not occur, and in their normal controls. It should be noted that C57BL/6 mice are known to be more difficult to immunize successfully with γ-spz (Doolan & Hoffman, 2000). These observations demonstrated that developmentally arrested hepatic parasites are not a pre-requisite to obtain sterile immunity, and that this phenomenon does not appear to be restricted to a particular genetic background.

Nonetheless, neither the absence of blood infection following s.c. inoculation nor sensitive PCR detection can unequivocally exclude that some priming could have occurred outside the skin via antigen or parasites (whole or degraded) that might have exited the inoculation site and disseminated systemically. We exploited the differences in the expected priming site following s.c. or i.d. inoculation (skin *versus* skin and systemic) to seek evidence of priming outside the skin. Subsequent to immunization with γ-spz, we measured the levels of primed T cells isolated from the lymph nodes draining the skin, the spleen or the liver, or after infection with wild type sporozoites. We further used an inhibitor of T lymphocyte migration and combined it with adoptive transfers of T cells specifically isolated from the various lymph nodes. All together the data strongly supported the hypothesis that sterile protection induced by s.c. immunization was primed predominantly, if not exclusively, in the skin-draining lymph node with little, if any, priming in the liver and/or the spleen.

Given the crucial role of DC in priming *Plasmodium*-specific CD8^+^ T cells in general and in the dermis in particular (Chakravarty et al, 2007), we exploited transgenic mice, whose CD11c^hi^ DC subsets can be depleted conditionally, to demonstrate that these cells play an essential role in the sterile immunity conferred by s.c. immunization. Moreover, we further demonstrated that adoptively transferred CD11c^hi^ DC incubated *ex vivo* with γ-spz are able to orchestrate the immunological events that lead to sterile protection. These observations provide solid evidence that the antigens responsible for induction of sterile immunity are present in the irradiated sporozoite. This might appear to contradict previous reports suggesting that protective antigens only appear in the hepatic parasite during the partial intrahepatocytic development stage (Frevert et al, 2005; Krzych et al, 2000). However, it is highly likely that the antigenic composition of the sporozoites altered after inoculation into the skin and prior to their acquisition by the skin-draining lymph node DCs. First, it has been shown that sporozoites could de-differentiate and initiate extracellular development into forms similar to the early hepatic stages (Kaiser et al, 2003). Second, the transition of sporozoites from the mosquito environment to that of the mammalian host cell is sufficient to induce the expression of genes normally transcribed in the early stages of the hepatic infection (Siau et al, 2008). Third, a proportion of the sporozoites deposited into the skin can develop into extra-hepatic pre-erythrocytic forms at the site of inoculation (Gueirard et al, 2010; Voza et al, 2010). Finally, the process of radiation attenuation might have led to the expression of genes normally only expressed in other stages including the maturing hepatic parasites (Hoffman & Chattopadhyay, 2008). Thus, in our model, the fact that EEFs were not detectable in the DC cultures following incubation with γ-spz, does not exclude a protective role for antigens normally expressed by hepatic stage parasites. The nature of the antigens responsible for the induction of the sterile immunity we observed merits further investigation.

The relevance of the data presented here is best appreciated in the context of efforts to develop an effective malaria vaccine targeting the pre-erythrocytic stages. The inability of the experimental subunit vaccines, including the leading formulation RTS,S, to confer sterile protection against infection has revived the concept of vaccination with live sporozoites, by they radiation-attenuated (Luke & Hoffman, 2003), genetically attenuated (Vaughan et al, 2010), or under the cover of an anti-blood stage drug (Belnoue et al, 2004, 2008). For these three strategies, the hepatic stage parasites that arise from the immunizing sporozoites clearly provide the most important source of antigens for the induction of the potent protective responses leading to sterile protection. Indeed, the immunizing hepatic forms substantially increase the diversity of antigens to which the immune system is exposed. Thus, to be effective, a whole sporozoite vaccine should ideally lead to the generation of as many hepatic forms as possible, from a number of immunizing sporozoites that would be practicable to produce and administer. In this context, the route of immunization becomes an issue of importance. The proportion of sporozoites that reach the liver is highest when they are inoculated naturally via a mosquito bite or i.v., and lowest when the i.d. or s.c. routes suitable for routine vaccination in humans is used. This might account for the contrasting efficacy observed in recent trials where two vaccination strategies were tested. Sterile protection against infected mosquito bite challenge was obtained in all ten volunteers immunized by the bite of 12–15 *P. falciparum*-infected mosquitoes on three monthly occasions while under chloroquine cover (Roestenberg et al, 2009). Given that a singly infectious bite delivers a median number of 15–22 sporozoites (Ponnudurai et al, 1991; Rosenberg et al, 1990), an estimated 1000 or so *P. falciparum* sporozoites were sufficient to achieve protection. By contrast, only 2 of 44 volunteers immunized by i.d. or s.c. inoculations with variable numbers and doses (7500 × 4 to 135,000 × 6) of purified cryopreserved irradiated *P. falciparum* sporozoites were fully protected against challenge with infectious mosquitoes (Epstein et al, 2011). The two protected individuals were in the group that received 120,000 irradiated *P. falciparum* sporozoites (divided equally over four doses). At this stage, the reasons for this failure remain speculative. The attenuating radiation dose is known to play an important part in the success of the immunization, in particular over-irradiation leads to a loss in the acquisition of protection, most probably by reducing the number of sporozoites that retain sufficient viability to reach and invade hepatocytes. Moreover, the influence of cryopreservation on the physical and biological integrity of the immunizing sporozoites might also play an adverse role. Disruption of the surface membrane of thawed cryopreserved sporozoites might have altered their recognition and processing by skin DC, led to increased mortality and rapid removal in the skin, or decreased their infectivity to hepatocytes. However, in the same study sterile protection was induced in mice immunized with a total of 6000 irradiated *P. yoelii* sporozoites (given in three equal doses) that were purified and cryopreserved in a similar manner to those from *P. falciparum*. For these experiments, as compared to the i.v. route, s.c. or i.d. immunization required 7–10 times more irradiated *P. yoelii* sporozoites to achieve the same levels of sterile protection in the mice (Epstein et al, 2011). Furthermore, for the same immunizing irradiated sporozoite dose, the number of specific CD8^+^ IFN-γ-producing T cells induced by the i.v. route was tenfold higher than that induced by i.d. route, an important consideration given the demonstration that long-term sterilizing immunity is rarely observed when memory CD8^+^ T cells fall below a certain threshold (Schmidt et al, 2008). The influence of the route of immunization was also noted for the *P. yoelii* parasite with a deleted *FabB/F* gene, a genetic attenuation that leads to developmental arrest of the late liver stages (Butler et al, 2011). Sterile protection could be induced by immunization with a total of 2000 sporozoites i.v., whereas 150,000 were needed by i.d. or s.c. administration.

Taken together, these observations raise the possibility that it might be technically challenging to develop live sporozoite immunization protocols whose efficacy relies on arrested hepatic stage development. Indeed, the i.v. route is the least practical for mass administration, while the production of the larger numbers of sporozoites needed for efficient s.c. or i.d. immunization might overwhelm capacity. Finally, it might be necessary to improve the viability and infectivity of the immunizing sporozoites subsequent to their cryopreservation and transport to immunization centers. We present here proof-of-concept that sterile protection can be obtained by s.c. immunization, a highly practical route of immunization in humans, and without exposure to hepatic stage parasites. However, to translate this potential into a deployable human vaccine, it will be necessary to identify the antigen, or antigens, that are responsible for the sterile immunity we observed, as well as the immunological events leading to the induction of this protection. Historically, observations on immunization against the pre-erythrocytic stages in rodent malaria models have translated to *P. falciparum* in humans. Although they do not offer optimal predictive pre-clinical vaccine models, they remain an excellent model to elucidate the immune mechanisms that could then guide vaccine development.

## MATERIALS AND METHODS

### Ethics statement

This study was carried out in strict accordance with the recommendations in the Guide for the Care and Use of Laboratory Animals of the European Union “European directive 86/609/EEC” and of the National Institutes of Health. The protocol was approved by the Committee on the Ethics of Animal Experiments of the University of Pierre et Marie Curie, Paris 6, France (Permit Number: A751301). All surgery was performed under sodium pentobarbital anaesthesia, and all efforts were made to minimize suffering.

### Animals and sporozoite preparations

Six- to eight-week-old female BALB/cJ mice were purchased from Janvier (France) and CD11c-DTR transgenic BALB/c mice were purchased from Jackson Laboratory (USA). C57BL/6 *Cd81*^−/−^ mice (back-crossed 12 times on the C57BL/6 background) were provided by Prof Shoshana Levy (Stanford, USA). Animal handling was conducted according to European regulations. Wild-type *Plasmodium yoelii yoelii* 265BY parasites were obtained by dissection of infected *Anopheles stephensi* mosquito salivary glands 2–3 weeks after their infective blood feeding on parasitized Swiss mice as described (Silvie et al, 2003).

### Mice infection and immunizations

In some groups, sporozoites *P. yoelii* 265BY were attenuated by exposure to 80 Gy of γ-radiation (^137^Cesium source at Necker Hospital, Paris, France). The dose of 80 Gy was sufficient to completely prevent the appearance of blood stage parasites in inoculated BALB/cJ mice even after the injection of high dose (100 × 10^3^) of irradiated sporozoites. BALB/cJ, C57BL/6 *Cd81*^−/−^ or *Cd81*^+/+^ mice (Silvie et al, 2003) were immunized either intradermally (i.d.), or subcutaneously (s.c.) using a total volume of 20 µl per dose in the lower flank, with three doses of PBS or γ-spz at weekly intervals, in some experiments with two weekly doses of 200 × 10^3^, or one dose of 600 × 10^3^ γ-spz, or i.v. by retro-orbital injection with 3-weekly doses of PBS or 50 × 10^3^ γ-spz (100 µl per dose). In some experiments, mice were immunized i.v., i.d. or s.c. with one dose of 75 × 10^3^ γ-spz followed by four doses of 20 × 10^3^ γ-spz at 2-weekly intervals. One week after the last immunization, mice were challenged i.d. (20 µl total volume) in the contralateral lower flank or i.v. (100 µl total volume) with 5 × 10^3^ live infectious sporozoites of the corresponding PyWT (*P. yoelii* 265BY), or naturally by infection through the corresponding mosquito bite by allowing a minimum of 10–20 wild-type infected female mosquitoes to bite on each mouse for 20 min. Blood-stage patency was monitored three times a week by evaluation of Giemsa-stained blood smears from day 4 to day 14 post sporozoites infection.

### Depletion of DC

We used BALB/c transgenic mice (CD11c-DTR) with a DT-based inducible system that allows the short-term ablation of DC *in vivo*, where ip injection of DT (Sigma–Aldrich), (4 ng/g) rapidly depletes (72–96 h) the DC subsets (Jung et al, 2002). CD11c-DTR mice can support multiple DC depletions, up to six repeated injections of DT (4 ng/g) 3 days apart, without loss of weight and mortality observed in C57BL/6 transgenic mice (Zammit et al, 2005; and data not shown). Two days after the depletion of DC, CD11c-DTR mice were immunized i.d. (20 µl) or s.c. (20 µl) with 200 × 10^3^ γ-spz for two boosters at weekly interval or 600 × 10^3^ γ-spz for one booster. This depletion was induced 2 days before each immunization two times by week. In some experiments, 1 week after the last immunization, mice were challenged i.d. (20 µl) with 5 × 10^3^ PyWT.

### Depletion of NK, CD8^+^ or CD4^+^ T cells and neutralization of interferon-γ

Mice were injected intraperitoneally with 5 mg/kg body weight of either an anti-CD4^**+**^ or anti-CD8^**+**^ neutralizing antibody (clone GK1.5 or clone 53–6.72, respectively; eBioscience), an isotype control antibody (BD Biosciences), an IFN-γ neutralizing antibody (clone XMG1.2; eBioscience), or with 25 mg/kg body weight of anti-asialo GM1 for NK cells depletion (Wako Bioproducts) 3 and 1 days before the s.c. immunization with γ-spz. Depletion of T-cell subsets and NK cells was confirmed by flow cytometry using splenocytes from immunized mice 1 week after the second injection. In some experiments, CD4^**+**^ or −CD8^**+**^ T cells were depleted 1 week after the last s.c. immunization with γ-spz and 3 days before the i.d. challenge with 5 × 10^3^ PyWT.

### Adoptive transfer

Five days after the second or the last i.v. or s.c. immunization with γ-spz (80 Gy), the spleen of BALB/c, C57Bl/6 *Cd81*^+/+^ or C57Bl/6 *Cd81*^−/−^ mice were extracted, and the splenocytes harvested after red blood cell lysis using with Red Blood Cell Lysis Buffer 1X (eBioscience) for 2–3 min at 4°C. A total of 30 × 10^6^ splenocytes were adoptively transferred into correspondent BALB/c or C57Bl/6 recipient naïve mice. Two days after adoptive transfer, the mice were challenged i.d. with 5 × 10^3^ PyWT. In some experiments, cells from the inguinal-draining lymph node (iLN), or the contralateral inguinal lymph node (nLN) of γ-spz s.c. immunized BALB/c, C57Bl/6 *Cd81*^+/+^, C57Bl/6 *Cd81*^−/−^ (treated or not with FTY720) or of CD11c-DTR mice injected with PBS or DT were adoptively transferred as detailed above.

### Drugs

FTY720 (Biovision) was injected intraperitoneally at a doses of 1 mg/kg every 24 h beginning at 1 day before the s.c. or i.d. immunization with γ-spz and during all the entire period of the experiment. The assessment of IFN-γ secretion, the challenge of s.c. vaccinated mice and the adoptive transfer of iLN, nLN or the spleen was conducted as previously described.

### *In vitro*-generated DC and infection

Dendritic cells were prepared as described (Naik et al, 2005), bone marrow cells were extracted from the tibias and femurs of BALB/c mice with culture medium composed of RPMI 1640 medium (Invitrogen Life Technologies) supplemented with 10% heat-inactivated FBS (Invitrogen Life Technologies), sodium pyruvate (Invitrogen Life Technologies), 50 µM 2-mercaptoethanol (Sigma–Aldrich), 10 mM HEPES buffer (pH 7.4) (Invitrogen Life Technologies), and 1% of penicillin/streptomycin (Invitrogen Life Technologies). After one centrifugation, BM cells were resuspended in Tris-ammonium chloride for 2 min to lyse RBC. After one more centrifugation, BM cells were cultured at 1 × 10^6^ cells/ml in culture medium supplemented with 100 ng/ml recombinant mouse FLT3 L (R&D systems) in six-well plates (Costar Corning). Cultures were incubated at 37°C in 5% CO_2_-humidified atmosphere. After 8–10 days, the non-adherent and loosely adherent cells were harvested with Versene, washed and transferred in 12-well plates (2 × 10^6^ cells/plate) for co-cultures (inoculation) with sporozoites of *P. yoelii* (PyWT or γ-spz) (2 × 10^4^). Sporozoites were incubated with the DC for 3 h and then cultures were washed and further incubated for the indicated period (0, 24 and 48 h) before EEFs quantification by immunofluorescence (see below) in triplicate wells.

### Hepatocyte preparation and infection

Primary mouse hepatocytes were isolated as described (Silvie et al, 2003), seeded in eight-chamber plastic Lab-Tek slides (Nalgene Nunc International) at a density of 8 × 10^4^ cells per well and cultured at 37°C in 4% CO_2_ in William's E medium (Gibco) with 10% foetal calf serum (Gibco), 2% penicillin–streptomycin (Gibco), 1% sodium pyruvate (Gibco) and 1% l-glutamine (Gibco), 1% insulin–transferrin–selenium (Gibco). Hepatocytes were cultured for 24 h before inoculation with PyWT sporozoites (2 × 10^4^), and then incubated for 3 h, at the end of which the cultures were washed and further incubated until the quantification of the EEFs by immunofluorescence (triplicate wells). In some experiments, we used the HepG2-A16-CD81 cell line that is highly receptive to *P. yoelii* sporozoites (Yalaoui et al, 2008).

### Immunofluorescence analysis

0, 24 or 48 h after infection, DC or primary mouse hepatocytes cultures were fixed with cold methanol and the EEFs were quantified by using a double-immunostaining technique and counted under a fluorescence microscope. Extracellular parasites were first labelled with a mouse monoclonal antibody directed against *P*. *yoelii* 265BY circumsporozoite protein (CSP) followed by a secondary antibody conjugated to phycoerythrin (PE). After DC or hepatocyte culture permeabilization with Triton X-100 1%, extra- and intracellular parasites were labelled with a polyclonal mouse serum directed against *P*. *yoelii* 265BY heat shock protein 70 (HSP-70) and revealed by a secondary antibody coupled to fluorescein isothiocyanate (FITC). DC, hepatocyte and parasite nuclei were stained with 1 µg/ml diamidinophenylindole (DAPI). Only parasites labelled with FITC (green) but not with phycoerythrin (red) were counted as intracellular (Renia et al, 1988; Silvie et al, 2003).

**The paper explained**

PROBLEM:

It has long been considered that the acquisition and maintenance of sterile immunity obtained through immunization with attenuated malaria sporozoites required the presence of infected hepatocytes. This is consistent with the failure of current experimental subunit vaccines against the pre-erythrocytic stages to prevent infection, which has led to renewed efforts in deploying vaccination with live attenuated sporozoites. Yet, a recent clinical trial of a skin-administered vaccine, based on cryopreserved *Plasmodium falciparum* radiation-attenuated sporozoites yielded very disappointing results, and led to suggest that success might necessitate i.v. inoculation, a major impediment to mass administration. A few years ago, it was shown that priming of protective immune responses following vaccination also occurs in the skin-draining lymph nodes, though the contribution of this to sterile immunity could not be determined. We wished to establish whether sterile protection following vaccination with radiation-attenuated sporozoites could be obtained even under conditions where few if any infected hepatocytes occur during immunization.

RESULTS:

We adopted two models, where hepatocyte infections are minimized following *P. yoelii* sporozoite inoculation. In the first model, this was done s.c. rather than i.d., and in the second when mice deficient in a molecule necessary for productive hepatocyte infection (CD81) were used. Using sterile protection as a read-out we showed that full protection could be obtained in both models by immunization with radiation-attenuated sporozoites. We further demonstrated that priming of the immune reactions leading to this sterile protection occurred predominantly if not exclusively through the dendritic cells in the skin-draining lymph nodes.

IMPACT:

The s.c. route of immunization is more practical for mass vaccination of humans. Our demonstration that priming in the skin with the antigens present in, or derived from, live attenuated sporozoites is sufficient to confer sterile protection provides a proof-of-principle for a new anti-infection vaccination strategy centred on skin-based immune responses. Furthermore, it opens the way to identify the antigen(s) that underlie the sterile protection induced, in the hope that this might lead to a subunit vaccine capable of preventing malaria infections. These observations may have implications for the vaccination field, based on manipulation of skin immunology and cutaneous biology.

### Transfer of DC

Dendritic cells were co-incubated at 37°C for 1.5 h with sporozoites. They were then sorted by FACS after CD11c staining (R&D systems) and 100 × 10^3^ were transferred to each of the recipient naïve BALB/c mice. One week after the transfer, the mice were challenged with infectious sporozoites and blood-stage patency was monitored three times a week by evaluation of Giemsa-stained blood smears from day 4 to day 14 following sporozoites infection.

### Assessment of interferon-γ production

BALB/c, *Cd81*^−/−^, *Cd81*^+/+^ (depleted for NK cells and treated or not with FTY720) or CD11c-DTR mice (injected ip with DT or PBS) were immunized s.c. (20 µl) with one dose of 200 × 10^3^ of γ-spz or PyWT. Five days later, spleen, iLN or nLN cells were recovered by homogenizing and filtering the organ through a sterile cell strainer (70 mm; Becton Dickinson). 1 × 10^5^ cells were cultured in complete culture medium in the presence or absence of the CSP epitope SYVPSAEQI in 200 µl medium in round-bottom 96-well plates. Three days later, the supernatants were harvested and IFN-γ secretion was determined by ELISA (eBioscience).

### Nested RT-PCR

A nested PCR protocol targeting the small subunit ribosomal RNA genes of the parasite offers a very high sensitivity: 1–10 target copies, or <10 parasites, per aliquot tested (Snounou & Singh, 2002). Given that RNA copy number/parasite is higher than that of DNA/parasite, the template for the nested PCR reaction was derived by reverse transcription from the purified RNA (RT-nPCR) in order to increase detection sensitivity. Mice were injected i.v., i.d. or s.c. with γ-spz or PyWT with the appropriate dose. Some mice were sacrificed after 40 h and their livers removed, or after 4 h and their spleens removed. Others mice inoculated s.c. with γ-spz were sacrificed 1, 6 or 12 h post-injection, and their spleens and livers removed. In other experiments, DC were FACS sorted immediately, 24 h, or 48 h following incubation with γ-spz. As a control, HepG2-A16-CD81 inoculated with γ-spz were harvested after washing 3 times with culture medium after 48 h of incubation. Total RNA was then purified from all the samples according to manufacturer's instruction (Kit PureLink™ micro-to-midi, Invitrogen) and all the work was conducted under RNase-free conditions. The total RNA was treated by DNase with DNA-free (Ambion-Pharmacia) and the concentration measured with NanoDrop (Thermo Scientific). Generally, about 45 µg were obtained from the liver, 15 µg from the spleen, and 15 µg from the DC. This RNA was reverse transcribed with the SuperScript® VILO™ cDNA Synthesis Kit (Invitrogen). The primers used for the PCR are specific to the small subunit ribosomal RNA (ssrRNA) of any *Plasmodium* parasite and were as follows: the external primers, rPLU1: 5′-TCAAAGATTAAGCCATGCAAGTGA-3′, rPLU2: 5′-ATCTAAGAATTTCACCTCTGACATCTG-3′; and the internal primers, rPLU3 5′-TTTTTATAAGGATAACTACGGAAAAGCTGT-3′, rPLU4: 5′-TACCCGTCATAGCCA-TGTTAGGCCAATACC-3′. The first round of PCR was carried out with rPLU 1 and 2 primers in a 20 µl volume containing 1× buffer, 1.5 mM MgCl2, 0.25 µM primer mix, 0.25 mM dNTP, 0.025 U/µl AmpliTaq polymerase (Applied Biosystems) and a cDNA aliquot derived from 30 ng of the starting total RNA. After initial denaturation at 95°C for 5 min, 25 cycles of DNA amplification were performed (25 cycles) of 62°C 1 min, 72°C 1 min, 94°C 30 s; then 62°C 1 min, 72°C 5 min and leave at 25°C. Subsequently, 1 µl of first round products was subjected to second round PCR for 35 cycles in 20 µl mixture containing 0.25 µM of each primer RPLU3 and 4. After amplification, 5 µl PCR products were electrophoresed in 1% agarose gel, stained with SYBR® safe, and photographed on an ultraviolet light transilluminator. The PCR product corresponds to a band of 231 bp. During the PCR process, strict precautions to minimize the cross contamination were adopted and numerous negative controls were included to monitor their effectiveness (Snounou & Singh, 2002).

### Statistical analysis

Experimental results are expressed as mean ± standard deviation (SD). Statistical significance was determined by Kruskal–Wallis test with Dunn's post-test. Statistics were performed with GraphPad InStat 3.

## Author contributions

MO designed experiments and performed most of the experiments; JFF also contributed to experiments and supervised insectarium activity; AL, GS and ACG. performed nested RT-PCR experiments and contributed to mosquitoes dissection; AG and MT contributed to some animal experiments and mosquitoes dissection; MT contributed to insectarium activity; CB produced the C57Bl/6 back-crossed *Cd81*^−/−^ mice; The experimental data were analysed and discussed by MO, GS, JFF and DM; MO and GS. wrote the manuscript. All authors commented and corrected the manuscript.

## Supplementary material

As a service to our authors and readers, this journal provides supporting information supplied by the authors. Such materials are peer reviewed and may be re-organized for online delivery, but are not copy-edited or typeset. Technical support issues arising from supporting information (other than missing files) should be addressed to the authors.
