# Supplementary material for: Skin-draining lymph node priming is sufficient to induce sterile immunity against pre-erythrocytic malaria
Source: EMBO Mol Med. 2012 Dec 19;5(2):250–63. doi: 10.1002/emmm.201201677 (PMC3569641; doi:10.1002/emmm.201201677)
Supplement: Supplementary file 1 [file emmm0005-0250-SD1.pdf]

Skin-draining lymph node priming is sufficient to induce sterile immunity against pre-erythrocytic malaria

Michel Obeid, Jean-Francois Franetich, Audrey Lorthiois, Audrey Gego, Anne Charlotte Grüner, Maurel Tefit, Claude Boucheix, Georges Snounou and Dominique Mazier

Corresponding author: Michel Obeid, Inserm UMR-S 945 and CHU Pitié-Salpêtrière, Paris

Review timeline:

	Submission date:	01 March 2012
	Accepted:	09 November 2012

Transaction Report:

No Peer Review Process File is available with this article, as the authors have chosen not to make the review process public in this case.

© EMBO
